# Accuracy of CT-Guided Core-Needle Biopsy in Diagnosis of Thoracic Lesions Suspicious for Primitive Malignancy of the Lung: A Five-Year Retrospective Analysis

**DOI:** 10.3390/tomography8060236

**Published:** 2022-11-25

**Authors:** Elisa Baratella, Stefano Cernic, Pierluca Minelli, Giovanni Furlan, Filippo Crimì, Simone Rocco, Barbara Ruaro, Maria Assunta Cova

**Affiliations:** 1 Department of Radiology, Cattinara Hospital, University of Trieste, 34127 Trieste, Italy; 2 Department of Medical Surgical and Health Sciences, University of Trieste, 34149 Trieste, Italy; 3 Institute of Radiology, Department of Medicine—DIMED, University of Padova, 35128 Padova, Italy; 4 Pulmonology Unit, Department of Medical Surgical and Health Sciences, University Hospital of Cattinara, University of Trieste, 34149 Trieste, Italy

**Keywords:** lung nodule, lung cancer, lung lesion, Core-Needle Biopsy (CNB), thoracic biopsy, percutaneous biopsy, diagnostic accuracy, sensitivity, specificity, Interventional Radiology (IR)

## Abstract

Background: Lung cancer represents a heterogeneous group of neoplasms, with the highest frequency and mortality in both sexes combined. In a clinical scenario characterized by the widespread of multidetector-row spiral CT, core-needle biopsy under tomographic guidance is one of the main and safest methods to obtain tissue specimens, even though there are relatively high rates of pneumothorax (0–60% incidence) and pulmonary hemorrhage (4–27% occurrence rates). The aim of this retrospective study is to assess the diagnostic accuracy of CT-guided core-needle biopsy in the diagnosis of primary lung malignancies and to compare our results with evidence from the literature. Materials and Methods: Our analysis included 350 thoracic biopsies, performed from 2017 to 2022 with a 64-row CT guidance and 16/18 G needles mounted on a biopsy gun. We included in the final cohort all samples with evidence of primary lung malignancies, precursor lesions, and atypia, as well as inconclusive and negative diagnoses. Results: There was sensitivity of 90.07% (95% CI 86.05–93.25%), accuracy of 98.87% (95% CI 98.12–99.69%), positive predictive value of 100%, and negative value of 98.74% (95% CI 98.23–99.10%). Specificity settled at 100% (93.84–100%). The AUC was 0.952 (95% CI 0.924–0.972). Only three patients experienced major complications after the procedure. Among minor complications, longer distances from the pleura, the presence of emphysema, and the lower dimensions of the lesions were correlated with the development of pneumothorax after the procedure, while longer distances from the pleura and the lower dimensions of the lesions were correlated with intra-alveolar hemorrhage. Immunohistochemistry analysis was performed in 51% of true positive cases, showing TTF-1, CK7, and p40 expression, respectively, in 26%, 24%, and 10% of analyzed samples. Conclusions: The CT-guided thoracic core-needle biopsy is an extremely accurate and safe diagnostic procedure for the histological diagnosis of lung cancer, a first-level interventional radiology exam for peripheral and subpleural lesions of the lung, which is also able to provide adequate samples for advanced pathologic assays (e.g., FISH, PCR) to assess molecular activity and genetic sequencing.

## 1. Introduction

Primary malignancies of the lung represent a heterogeneous group of neoplasms with the highest mortality and frequency in men and women combined [1], with age-standardized incidence and mortality of, respectively, 22.4 and 18.0 per 100,000 people [2]. Even though its incidence in the Western world has been constantly declining since the mid-1980s of the last century, in 2022, about 237,000 new diagnoses and 130,000 deaths are estimated in the US (69,000 men, 31,000 women). Most of the lung cancers are classified as non-small cell lung cancer (NSCLC, 82%) and small cell lung cancer (SCLC, 14%) [3]. The main risk factors for the development of the disease are represented by cigarette smoking (80–90% of affected patients have a positive history of smoking), either active or secondhand, radon gas, aging, and environmental or occupational exposure to carcinogenic agents (at least 10% of total cases of lung cancers present a previous history of occupational exposure) [1,2,3,4]. Most patients come to clinical and diagnostic evaluation because of suspicious symptoms or incidental abnormal findings on chest imaging. Nowadays, several different techniques are available in order to determine a definitive diagnosis: selecting a specific diagnostic modality should maximize the diagnostic yield and avoid distressful and unnecessary tests for the patient. FDG-PET/CT combines both conventional and metabolic imaging [5]. This hybrid modality detects lesions with an avid glucose metabolism, a feature highly consistent with malignant lesions; however, some indolent lung cancers may demonstrate a weak metabolic activity, while a wide spectrum of flogistic non-neoplastiv conditions could show a high FDG captation. Sputum cytology is the least invasive diagnostic modality, and its main indications are for central lesions (mainly squamous cell cancers) and bloody sputum, but its accuracy is widely influenced by sputum collection. Flexible broncoscopy (FB) forcep biopsy shows a high diagnostic yield for central lesions, but its sensitivity for peripheral cancer is considerably lower; radial EBUS (R-EBUS) is an adjunct imaging modality for suspicious peripheral lung nodules, especially in patients for whom surgery is not indicated [6], and it brings real-time ultrasound images in order to better assess the ideal location of sampling. Among surgical techniques, small solid peripheral nodules are amenable for video-assisted thoracoscopy (VATS) [7] and, along with open biopsy, provide the largest pathology specimens. However, surgical methods are inevitably associated with higher rate of complications and longer post-operative recovery. Percutaneous image-guided biopsy of the lung (with ultrasound, tomographic, and/or fluoroscopic guidance) allows to carry out microhistological and cytological samples using minimally invasive procedures, resolves diagnostic suspicion, and early addresses the diagnostic-therapeutic path, resulting in a reduced discomfort for the patient and a significant decrease in frequency and entity of intra- and periprocedural complications, in comparison with surgical biopsy techniques [8,9]. In the era of personalized and precision medicine, constant improvements in molecolar tissue characterization and in pathologic assessment techniques broadly speaking (e.g., immunohistochemistry staining and direct gene sequencing), along with progression in imaging techniques, have led to a considerable growth of indications for percutaneous biopsy. Nowadays, these procedures are also performed to assess disease progression and prognosis and to eventually provide personalized therapy. The future will, therefore, rely more on biopsies, with the consequent need for more adequate cytologic and histologic samples [10,11,12]. The main limitation of transthoracic biopsy procedures is, however, represented by the high relative rate of some complications: among these, pneumothorax (PNX) is the most common, showing an incidence up to 60% and an average risk of about 20%, even though chest tube placement is required with a reported rate of only 1 to 14% [13,14,15]. Several risk factors are associated with pneumothorax, such as lesion size and depth, experience of radiologist, number of pleural punctures, non-perpendicular needle angle to pleura, and the imaging evidence of chronic obstructive pulmonary disease [14,16,17]. Pulmonary hemorrhage (PH) is the second most frequent complication: it can be identified as perilesional ground-glass opacity and shows occurrence rates from 4 to 27%, with hemoptysis risk up to 5%. Bleeding is proved to be more frequent when the lesion is <2 cm and the needle path > 4 cm [14,18]. Other rare complications are systemic air embolism and tumor seeding (reported rates of 0.06% and 0.01–0.06%, respectively) [15]. The aim of this single-center, observational retrospective study is to evaluate the diagnostic accuracy of CT-guided core-needle biopsy (CNB) in the diagnosis of primary malignant neoplasms of the lung and to compare the results with the evidence from the most accredited state-of-the-art literature.

## 2. Materials and Methods

This retrospective study conformed to the principles of the Declaration of Helsinki for medical research, according to the local diagnostic-therapeutic-assistance path (DTAP) for the management of lung nodules. From January 2017 to January 2022, 490 patients underwent at least one core-needle thoracic biopsy. Among these, 410 were admitted to the interventional radiology (IR) division with the pre-eminent diagnostic suspicion of primary malignancy of the lung. Patients were eligible for study inclusion if they met the following criteria: (i) execution of the biopsy procedure, even in cases of partial technical success and/or intra-procedural complications, either minor or major (e.g., massive hemoptysis); (ii) presence of solitary nodules of the lung and target masses not exceeding 50 mm in size (measurement of the long axis of each lesion), because these lesions likely demonstrate necrosis, with a consequent impairment of diagnostic accuracy and a higher rate of false negatives [14]; (iii) diagnosis of primary malignant neoplasm of the lung, atypical alveolar hyperplasia, and other precursor lesions (e.g., squamous dysplasia); (iv) microhistological samples characterized by non-specific findings (inflammation, necrosis, and fibrosis), as well as biopsies “uncertain for malignant disease”; and (v) negative diagnosis for lung cancer. Our retrospective analysis, therefore, excluded patients with the following criteria: (i) procedure not executed, i.e., technical failure; (ii) target masses exceeding 50 mm in long axis; (iii) microhistological diagnoses different from the ones mentioned in the inclusion criteria (e.g., pulmonary localization of secondary neoplasm, rheumatoid nodules, etc.); (iv) patients for whom lung cancer was not the indication for the procedure; and (v) all biopsies performed under cone-beam CT (CBCT) and ultrasound (US) guidance (Table 1). Other diagnostic techniques, such as endoscopic techniques, were excluded because of the peripheral location of the lesions that underwent transthoracic biopsy—therefore not meeting the indication for surgical or endoscopic methods—or because, when already executed, lead to inconclusive results.

The results of biopsy sampling were evaluated in relation to the definitive surgical specimen, when available, or the clinical-radiological follow-up: this post-procedure assessment constitutes the double end point of our study. The risk for patients of being affected by lung cancer was defined by anamnestic and clinical data, by pre-procedure imaging (either radiological or nuclear), and by the discussion about the indication for the procedure for single clinical cases in the multidisciplinary team (lung cancer unit) with fellow pulmonologists, thoracic surgeons, nuclear medicine physicians, radiotherapists, oncologists, and pathologists.

### 2.1. CT Acquisition Protocol

As aforementioned, all biopsy procedures were performed under CT guidance. Procedural CT scan was performed with a 64-row MDCT system (Aquilion 64, Toshiba Medical Systems, Tokyo, Japan). The technical parameters were a rotation time of 500 ms; a tube voltage of 120 kV; a tube current (effective mA) of 150–400, depending on the patient’s size, with modulated exposure to reduce the dose (Sure exposure: standard); a field of view of 40 cm; and a reconstruction thickness of 2 mm with a reconstruction interval of 2 mm; the CT acquisition has always been volumetric to allow for any multiplanar reconstructions to be performed. CT scans were limited to the lesion area with a margin of a few cm to limit patient exposure; only the final scan was performed involving a greater portion of the lung to better evaluate post-procedure complications.

### 2.2. Biopsy Technique

For each patient, the fitness for the procedure was assessed and relative and absolute contraindications were excluded, especially of coagulative nature in compliance with CIRSE and SIR guidelines: in particular, complete blood count and coagulation profile were available for each patient, with platelet count > 50,000/μL and international normalized ratio (INR) ≤ 1.5 as necessarily required values for performing the procedure. Moreover, we considered an activated partial thromboplastin time (aPTT) value ≤1.5 as the mandatory cut-off for patients under endovenous fractionated heparin (LMWH). P2Y12 inhibitors and direct oral anticoagulants (DOACs) were, respectively, withheld 5 days and 2–3 days prior to the biopsy, while LMWH was suspended a dose before the procedure [19,20,21]. Informed consent was acquired, providing full and clear information about indications and benefits of the procedure, as well as about risks and complications (with particular reference to pneumothorax, hemorrhage, and hemoptysis, as well as the possibility of chest tube placement) in observance to the national laws and forms of our institution [22].

**Figure 1:**
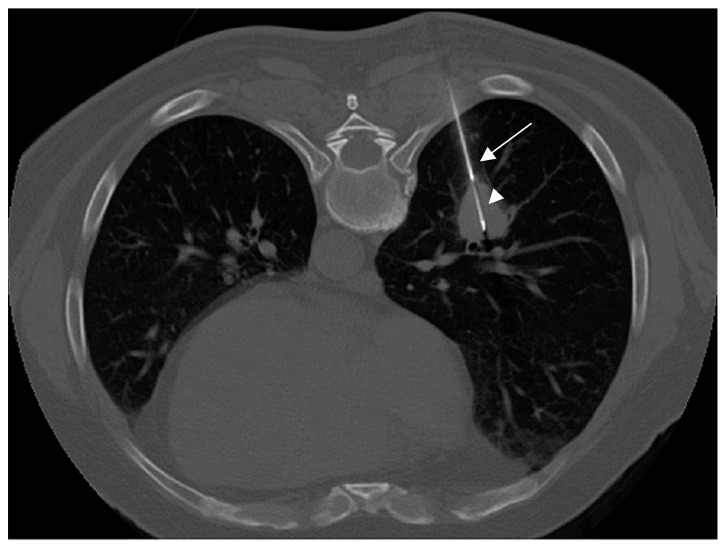
Core-needle biopsy of a central solid nodule of the lower left lobe; the extremity of the cutting needle appears in the center of the lesion (arrowhead), while the 17 G coaxial introducer needle was placed adjacent to the lesion (arrow). Pathological diagnosis demonstrated a moderately differentiated squamous cell carcinoma.

A localizing radiopaque grid was applied to the skin of each patient, following the evaluation of prior exams and immediate pre-procedure scans, in order to accurately identify and plan the access site. Local anesthetic (lidocaine 2%, 10 mL) was then administered at the designated site. The biopsy technique used was preferably the coaxial technique, with a 17 G introducer needle and an 18 G cutting needle mounted on a full-core needle biopsy gun; in a minority of cases of large superficial or mediastinal masses, 15 G introducer needles and 16 G cutting needles were used (Figure 1). This type of biopsy instrument made it possible to obtain one or more cylindrical tissue fragments with lengths from 13 to 33 mm.

### 2.3. Statistical Analysis

Sensitivity, specificity, positive and negative predictive value (PPV and NPV), and diagnostic accuracy were calculated and compared by keeping surgical pathology specimen and/or the clinical-radiological follow-up as the reference standards. Predictive values and diagnostic accuracy were dependent on the disease prevalence. These results were reported as mean ±95% confidence interval (95% CI), while data concerning the characteristics of the population were calculated as mean. Therefore, a “true positive” was defined as a CNB specimen leading to final diagnosis of primary lung malignancy, confirmed by a surgical specimen or by the subsequent clinical/radiological follow-up. A “true negative” was defined as a negative CNB specimen or with no evidence of malignant cells, with a non-malignant final diagnosis. We considered as “false negative” each biopsy specimen with no evidence of malignancy, with a final diagnosis of lung cancer obtained by surgical biopsy. A receiver operating characteristic (ROC) curve was drawn using the DeLong technique and the area under ROC curve (AUC) was calculated in order to evaluate the accuracy of CNB in the detection of malignancy, using the final diagnosis as a reference standard.

A stepwise backward logistic regression analysis, using 0.05 and 0.10 as inclusion and exclusion criteria, respectively, was used to identify the predictors of concordance between CNB findings and final diagnosis. The variables included in the initial model were the age of the patients, the distance of the lesions from the pleura, the maximum diameter of the lesions, and the number of samples collected with CNB.

Similar stepwise backward logistic regression analyses were also conducted to identify the predictors of four complications after CNB (pneumothorax, intra-alveolar haemorrhage, hemoptysis, and cardiopulmonary arrest). The variables included in the initial model were the following: the age of the patients, the presence of emphysema, the presence of fibrosis, the distance of the lesions from the pleura, the maximum diameter of the lesions, and the number of samples collected with CNB.

SPSS for Mac (version 27 for Mac, IBM-SPSS Bologna, Bologna, Italy) and MedCalc (MedCalc Software, Ostend, Belgium, version 15.8) were used for the statistical analysis.

## 3. Results

### 3.1. Study Population Characteristics

A total of 350 patients were finally enrolled, including 132 women (37.7%) and 218 men (62.3%). The mean age at the procedure is 72 years. A total of 199 procedures involving the right lung were performed (56.9% of total biopsies); among these, 124 (62.3%) involved the upper lobe (RUL), 13 (6.5%) the middle lobe (ML), 59 (29.7%) the lower lobe, while only 3 (1.5%) biopsies were executed on the pleura of the right lung.

Instead, 149 procedures involving the left lung were completed (42.5% of all the procedures); among these, 90 (60.4%) were performed on the upper lobe (LUL), 8 (5.4%) on the lingula (LIN), 48 (32.2%) on the lower lobe (LLL), and 3 (2%) were performed on the pleura of the left lung.

Only 2 biopsies targeted the mediastinum (0.6% of total biopsies).

### 3.2. CT Findings

**Figure 2:**
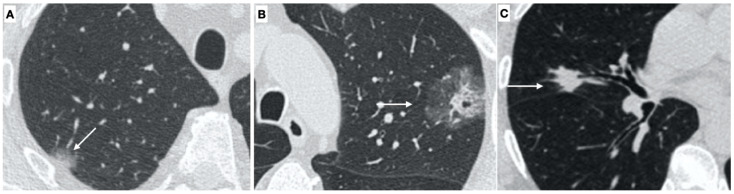
(**A**) High resolution CT scan shows a ground glass opacity in the right upper lobe (arrow); a minimally invasive adenocarcinoma with predominant lepidic growth was diagnosed. (**B**) High resolution CT scan demonstrates a partial solid nodule in the upper left lobe, with a solid component of 9 mm (arrow) and a final diagnosis of adenocarcinoma. (**C**) A solid nodule of 12 mm in the right upper lobe with (arrow) a final diagnosis of squamous cell carcinoma.

The biopsied lesions had diameters ranging from 9 to 50 mm (mean diameter 40 mm). Among these, 270 (77.1%) were solid density nodular formations, while 70 (20%) and 10 (2.9%) were, respectively, part-solid and ground-glass abnormalities. The morphology of the target lesions was thus distinguished between “rounded” and “non-rounded”: 158 abnormalities (45.1% of all findings) were classified as rounded, while 192 (54.9%) were described as non-rounded. We also reported the margins of the target lesions: 256 (73.1%) showed irregular margins, 38 (10.9%) had lobulated margins, 35 (10%) demonstrated smooth margins, while 21 (6%) showed ill-defined margins (Figure 2).

Relationships between the lesions and adjacent anatomical structures were assessed, and 80 (22.9%) showed pleural attachment: between these, 8 (10%) showed relationships with peripheral bronchial branches, 3 (3.75%) were in contact with hilar and peribroncovascular structures, while 1 (1.25%) demonstrated encasement of the subpleural adipose tissue. Two hundred seventy (270, 77.1%) lesions had a peripheral location in the lung parenchyma, however, having no relationship with the anatomical structures placed in proximity.

A total of 21 nodules subjected to core-needle biopsy (6% of the total) had calcifications, while 14 (4% of the total) were cavitary (Table 2).

### 3.3. Complications

After CNB, out of 350 patients, the majority showed only minor complications, respectively, i.e., 146 (41.7%) pneumothorax, 101 (28.9%) intra-alveolar hemorrhage, and 26 (7.4%) hemoptysis. Three patients experienced major complications after the procedure, two of those with hemoptysis had a cardiopulmonary arrest, recovered with the intervention of the anestesiologists, while one of the patients with intra-alveolar hemorrhage had a hemothorax and, therefore, was addressed to thoracic surgeons for drainage.

We investigated the prognosticators of pneumothorax, hemorrhage, hemopthysis, and cardiopulmonary arrest after CNB using a stepwise backward logistic regression analysis by including in the initial model all the most plausible predictors (age of the patients, the presence of emphysema, the presence of fibrosis, the distance of the lesions from the pleura, the maximum diameter of the lesions, and the number of samples collected with CNB).

For pneumothorax, collectively, the distance from the pleura (coefficient = 0.57081; *p* = 0.0120), the presence of emphysema (coefficient = 0.48439; *p* = 0.0417), and the lesion’s maximum diameter (coefficient = −0.01950; *p* = 0.0403) predicted 60.6% of the variance of the outcome variable (chi-squared = 14.715; *p* = 0.0021, Cox and Snell R^2^ = 0.04117).

For intra-alveolar hemorrhage, the distance from the pleura (coefficient = 0.97139; *p* = 0.0001) and the lesion’s maximum diameter (coefficient = −0.02978; *p* = 0.0056) together predicted 69.7% of the variance of the outcome variable (chi-squared = 27.300; *p* < 0.0001, Cox and Snell R^2^ = 0.07504).

For hemoptysis and cardiopulmonary arrest, no variables were retained in the model.

### 3.4. Microhistological Evidences

Epithelial neoplasms were the most frequently diagnosed in 253 microhistological samples out of 350 (72.3%). The most common histotype was represented by adenocarcinoma, found in 182 biopsy samples (52% of the total), followed by 45 diagnoses of squamous cell carcinoma (12.9%) and 16 cases of neuroendocrine tumor (NET, 4.5%), of which 6 (37.5% of total NET diagnoses) were identified as small cell carcinomas (former microcytomas). In 10 cases (2.9%), a more generic diagnosis of poorly differentiated carcinoma or non-small cell lung cancer (NSCLC, non-small lung cell carcinoma) was made, due to the inability to better define the histotype of the pathology specimen. In six cases (1.7%), the presence mesenchymal neoplasm was found.

In 20 cases (5.7%), the histological outcome was uncertain or inconclusive. In this category, we included all the cases that, despite the absence of neoplastic cells, reported cellular atypia, dysplastic/metaplastic elements, as well as the presence of atypical alveolar hyperplasia, which is a known precancerous condition, whose evolutionary potential in adenocarcinoma, however, has not yet been fully defined. Among this group, 12 patients (60% of the cohort) underwent surgical biopsy (either VATS or open) and were all diagnosed with definitive primary lung neoplasm. A total of 71 samples (20.3% of the total) were negative for neoplasm, precursor lesions, or atypia: 21 (29.6%) patients, therefore, underwent diagnostic completion by surgical biopsy and 16 (22.5% of the examined cohort) received a definitive diagnosis of lung cancer (Table 3).

### 3.5. Sensitivity, Predictive Values and Diagnostic Accuracy

The analysis found 263 true positives (TP, 75.1%), 58 true negatives (TN, 16.6%), 29 false negatives (FN, 8.3%), and 0 false positives (FP). The adequacy of the sample was reported by the pathologist as “suboptimal” in 6 out of 29 cases of false negatives (20.7%), since the amount of tissue was defined as not adequate for yielding a definitive pathological diagnosis, thus requiring further invasive procedures. In 134 out of 263 positive histological samples (51%), somatic genetic mutations and molecular expression were successfully detected using alternatively immunohistochemistry (IHC), polymerase chain reaction (PCR), and fluorescence in situ hybridization (FISH). Among this cohort, 35 samples (26.1%) demonstrated TTF-1 positivity, followed by 32 cases (23.9%) showing CK7 expression. P40 expression—which is highly specific for squamous cell differentiation [23]—was detected in 13 cases (9.7%). EGFR mutations were demonstrated in 10 samples (7.5%), while KRAS gene mutations were found in 7 cases (5.2%). There was sensitivity of 90.07% (95% CI 86.05–93.25%), accuracy of 98.87% (95% CI 98.12–99.69%), positive predictive value of 100%, and negative value of 98.74% (95% CI 98.23–99.10%). Specificity settled at 100% (93.84–100%). The calculated AUC was 0.952 (95% CI 0.924–0.972; *p* < 0.001) (Table 4).

We also investigated the prognosticators of concordance between CNB results and final diagnosis using a stepwise backward logistic regression analysis by including in the initial model: age of the patients, the distance of the lesions from the pleura, the maximum diameter of the lesions, and the number of samples collected with CNB. The number of samples (coefficient = 0.61970; *p* = 0.0644) predicted 92% of the variance of the outcome variable with border-line levels of significance (chi-squared = 3.720; *p* = 0.0538, Cox and Snell R^2^ = 0.01057).

## 4. Discussion

Primary malignant lung neoplasms constitute a heterogeneous nosological entity, however, characterized by a high frequency in the population, a generally poor prognosis (despite the most recent developments in the immunotherapeutic field and in target therapies), and high social costs. In the last two decades, technical implementations in diagnostic imaging, along with the continuous development of materials and techniques in biopsies and extravascular interventional radiology broadly speaking, have contributed to ease early diagnosis and to perform minimally invasive procedures with high diagnostic power. In this perspective, our study confirms the encouraging results about the diagnostic power of core-needle biopsy: the overall values of sensitivity and specificity are in line with what has already been reported in the literature [9,14,24] and account for the widespread use of CNB as a first-choice invasive procedure in the histological typing of the solitary pulmonary nodule, in particular for solid and part-solid nodules. The salient evidence, however, is represented by the very high diagnostic accuracy found (close to 99%), superior to what is already known [9,14,15,24,25]: this result may be justified by the dependence of the biopsy outcome on the expertise of the interventionalist [9,16]. Our center, in fact, presents high volumes of CT-guided percutaneous biopsies, with at least one dedicated weekly session, and all the analyzed procedures were performed by radiologists with at least fifteen years of experience in the extravascular and biopsy interventional field. The experience of the performing radiologist and the confidence with the procedure may decrease sampling errors, resulting in potentially less needle adjustments, a higher number of needle passes for each single lesion, and, therefore, an increased availability of material for histological sampling and a higher diagnostic efficacy [14,26]. The discussion about the indication for the procedure for single clinical cases in the multidisciplinary team lead to an accurate selection of patients undergoing the procedure, thus contributing to a high overall diagnostic power.

The peripheral and subpleural localization of all the lesions reaffirms and limits the indication to the procedure to the pulmonary cortex, for which it represents the first-choice biopsy procedure, due to a diagnostic performance, in this site, by far superior to bronchoscopy sampling methods and by a minimally invasive path [27,28].

The results concerning an accurate histological typing, along with the ability to characterize the degree of cell differentiation and the patterns of growth and invasiveness in most positive samples does confirm the high diagnostic power of the method in cases of adequacy of the sample [29]. Another piece of crucial evidence is represented by the possibility of performing both genomic analysis and molecular profiling on the sample: this retrospective analysis further confirms the central role of percutaneous biopsy in the complex setting of personalized medicine and target therapies that took over oncology in the last decade [11,30].

Although CNB has been constantly substituting more invasive approaches for histological diagnoses for the last decade [31], also taking into account the aforementioned evidence, in our study, the same procedural limitations already found in the previous bibliography emerge. Above all, the main issue is represented by the very limited quantity of the specimen material, often minimal if compared to the entire lesion and sometimes not representative of the real histology or inadequate for yielding diagnosis [9]. The amount of available tissue also depends on individual patient characteristics (habitus, respiratory fitness, and eventual presence of diffuse abnormalities of the lung parenchyma and interstitium) and by intra-procedural factors (minor and major complications that affect both technical and clinical outcomes). It is, therefore, not surprising that 37 cases (10.6% of the total population) led to an initial misdiagnosis, successively corrected by radical and invasive surgical interventions, in which, however, the histological specimen consists of the entire lesion and its borders.

The limitations of our study are represented by its retrospective nature, by its monocentricity, and by the loss of patients during the follow-up (21 patients, 6% of the total). A prospective controlled and multicentric study would allow the enrollment of a larger population, limiting the loss of patients after the first diagnosis and allowing a more accurate assessment of the diagnostic performance of the procedure. Moreover, it would provide a more detailed comparison among the great variety of different intra-procedural imaging protocols (e.g., standard-dose vs. low- and ultra-low-dose) about image quality and radiation safety, as well as technical outcomes for lesions of different size and density and overall diagnostic power. A prospective study may also provide further assessment concerning expected outcomes between the only core-needle biopsy versus combined FNAB-CNB procedure. In this regard, it is already known that a combined fine-needle aspiration and core-needle biopsy approach would further increase the diagnostic accuracy, providing cytomorphological information to be integrated with the evaluation of tumor architecture evaluable by the microhistological sample [11,32,33].

## 5. Conclusions

Our study confirms the evidence of the literature: CT-guided core-needle biopsy of the chest is an extremely accurate diagnostic procedure for the histological diagnosis of lung cancer, a first-level interventional radiology exam for peripheral and subpleural lesions, which is able to provide adequate material for immunohistochemistry. In the era of target therapies and cancer immunotherapy, it assumes even greater importance in the patient’s diagnostic-therapeutic process. Despite its slightly simple feasibility, further studies are, however, needed in order to implement the procedure, by comparing different radiation dose protocols and by deeply assessing differences between biopsy methods.

## Figures and Tables

**Table 1:** Inclusion and exclusion criteria.

Inclusion Criteria	Exclusion Criteria
Execution of the biopsy procedure	Procedure not executed/technical failure
Lesions ≤ 50 mm in size	Target masses > 50 mm diameter
Diagnosis of lung cancer, precursor lesions, and atypia	Microhistological diagnoses different from the ones in the inclusion criteria
Microhistological samples characterized by non-specific findings	Patients for whom lung cancer was not the indication for the procedure
Negative or inconclusive diagnoses	Biopsies performed under CBCT and US guidance

**Table 2:** Population characteristics and radiological features of the lesions.

Population Charasteristics	
Male patients	218 (62.3%)
Female patients	132 (37.7%)
Mean age at biopsy	72
Density	
Solid density nodules	270 (77.1%)
Part-solid nodules	70 (20%)
Ground-glass nodules	10 (2.9%)
Morphology	
Rounded	158 (45.1%)
Non-rounded	192 (54.9%)
Margins	
Irregular/spiculated	256 (73.1%)
Lobulated	38 (10.9%)
Smooth	35 (10%)
Ill-defined	21 (6%)
Relationships with adjacent structures	
Pleural attachment	80 (22.9%)
Peripheral, isolated lesion	270 (77.1%)
Other radiological features	
Calcified lesions	21 (6%)
Cavitary lesions	14 (4%)
Non-calcified, non-cavitary lesions	315 (90%)
Total biopsies	350

**Table 3:** Microhistological evidence from transthoracic biopsy sampling.

Histological Pattern	Number of Samples	Percentage (%)
Adenocarcinoma	182	52%
Squamous cell carcinoma	45	12.90%
Neuroendocrine tumor	16	4.50%
Non-subtyped NSCLC	10	2.90%
Mesenchymal neoplasm	6	1.70%
Uncertain/inconclusive	20	5.70%
Negative samples	71	20.30%
TOTAL	350	100%

**Table 4:** Sensitivity, specificity, predictive values, and diagnostic accuracy.

	Value	CI 95%
Sensitivity (%)	90.07	86.05–93.25
Specificity (%)	100	93.84–100
Positive Predictive Value (%)	100	
Negative Predictive Value (%)	98.74	98.23–99.10
Accuracy (%)	98.87	97.12–99.69
AUC	0.952	0.924–0.972

## Data Availability

The data presented in this study are available on request from the corresponding author.

## Abbreviations

CT: computed tomography; CNB: core-needle biopsy; CBCT: cone-beam CT; DOACs: direct oral anticoagulants; US: ultrasound; FNAB: fine-needle aspiration biopsy; INR: international normalized ratio; IR: interventional radiology; LMWH: low molecular weighted heparin; MDCT; multi-detector computed tomography; NPV: negative predictive values; PPV: positive predictive values; IHC: immunohistochemisty; PCR: polymerase chain reaction; FISH: fluorescence in situ hybridization.
